# Comparação de Soluções Cardioplégicas em Cirurgia de Revascularização Miocárdica sobre Mecanismos de Autofagia e Apoptose

**DOI:** 10.36660/abc.20220479

**Published:** 2023-06-28

**Authors:** Elif Funda Sener, Zuhal Hamurcu, Serpil Taheri, Reyhan Tahtasakal, Nesrin Delibasi, Deniz Elcik, Ecmel Mehmetbeyoglu, Aydin Tuncay, Fatma Dal, Keziban Korkmaz Bayram, Isın Gunes, Omer Naci Emirogullari

**Affiliations:** 1 Erciyes University Medical Faculty Department of Medical Biology Kayseri Turquia Erciyes University Medical Faculty Department of Medical Biology, Kayseri – Turquia; 2 Erciyes University Genome and Stem Cell Center Kayseri Turquia Erciyes University Genome and Stem Cell Center (GENKOK), Kayseri – Turquia; 3 Cappadocia University Cappadocia Vocational College Department of Medical Laboratory Techniques Nevsehir Turquia Cappadocia University Cappadocia Vocational College Department of Medical Laboratory Techniques, Nevsehir – Turquia; 4 Erciyes University Medical Faculty Department of Cardiology Kayseri Turquia Erciyes University Medical Faculty Department of Cardiology, Kayseri – Turquia; 5 Erciyes University Medical Faculty Department of Cardiovascular Surgery Kayseri Turquia Erciyes University Medical Faculty Department of Cardiovascular Surgery, Kayseri – Turquia; 6 Ankara Yildirim Beyazit University Medical Faculty Department of Medical Genetics Ankara Turquia Ankara Yildirim Beyazit University Medical Faculty Department of Medical Genetics, Ankara – Turquia; 7 Erciyes University Medical Faculty Department of Anesthesiology and Reanimation Kayseri Turquia Erciyes University Medical Faculty Department of Anesthesiology and Reanimation, Kayseri – Turquia

**Keywords:** Doença Arterial Coronariana, Revascularização Miocárdica, Isquemia Miocárdica, Soluções Cardioplégicas, Ponte de Artéria Coronária, Autofagia, Apoptose

## Abstract

**Fundamento:**

A doença arterial coronariana (DAC) devido à isquemia miocárdica causa perda permanente de tecido cardíaco.

**Objetivos:**

Nosso objetivo foi demonstrar o possível dano ao miocárdio em nível molecular através dos mecanismos de autofagia e apoptose em pacientes submetidos à cirurgia de revascularização miocárdica.

**Métodos:**

Um grupo recebeu uma solução de cardioplegia Custodiol e o outro grupo uma solução de cardioplegia sanguínea. Duas amostras miocárdicas foram coletadas de cada paciente durante a operação, imediatamente antes da parada cardíaca e após a liberação do pinçamento aórtico. Foram avaliadas as expressões de marcadores de autofagia e apoptose. O nível de significância estatística adotado foi de 5%.

**Resultados:**

A expressão do gene BECLIN foi significativa nos tecidos miocárdicos do grupo CS (p=0,0078). Os níveis de expressão dos genes CASPASE 3, 8 e 9 foram significativamente menores no grupo CC. Os níveis pós-operatórios de TnT foram significativamente diferentes entre os grupos (p=0,0072). As expressões dos genes CASPASE 8 e CASPASE 9 foram semelhantes antes e depois do pinçamento aórtico (p=0,8552, p=0,8891). No grupo CC, os níveis de expressão gênica de CASPASE 3, CASPASE 8 e CASPASE 9 não foram significativamente diferentes em amostras de tecido coletadas após pinçamento aórtico (p=0,7354, p=0,0758, p=0,4128, respectivamente).

**Conclusões:**

Com nossos achados, acreditamos que as soluções CC e CS não apresentam diferença significativa em termos de proteção miocárdica durante as operações de by-pass.

## Introdução

As soluções de cardioplegia são usadas para parar o coração e reduzir o dano isquêmico no miocárdio quando o fluxo sanguíneo coronário é interrompido durante as operações cardíacas.^1 , 2^ Como o Custodiol e a cardioplegia sanguínea são soluções altamente eficazes, elas têm sido usadas há muito tempo em cirurgias de coração aberto. Durante a circulação extracorpórea (CEC), o coração é parado e protegido com soluções de cardioplegia. Este período está associado à privação de oxigênio; o coração fica isquêmico durante a CEC. O coração é reperfundido ao final da CEC e a ação cardíaca continua.^3^ No entanto, a CEC pode levar a danos adicionais ao miocárdio causados por reperfusão global e morte celular por meio de indução de autofagia miocárdica e apoptose.^4^

Acredita-se que a autofagia desempenhe um papel absoluto no tecido cardíaco durante a isquemia/reperfusão. Enquanto a indução de autofagia em cardiomiócitos durante a isquemia tem um efeito protetor, acredita-se que a autofagia induzida durante a reperfusão leve à morte dos cardiomiócitos. Em condições normais, o estresse metabólico normalmente estimula a apoptose. No entanto, em células onde o mecanismo de apoptose é prejudicado, a célula continua a viver em condições hipóxicas. A sobrevivência dessas células se deve à autofagia, mas quando a apoptose e a autofagia são suprimidas, a viabilidade celular falha e morre.^5^ A autofagia ocorre em níveis basais, mas pode ser ainda mais induzida por estresse, como hipóxia e depleção de nutrientes.^6^ A proteína LC3B é um jogador importante no processo autofágico. A ativação da autofagia é refletida por aumentos na abundância de proteínas-chave dos genes relacionados à autofagia (ATG5-12), cadeia leve 3 (LC3), Beclin-1 e p62.^7^ As regiões miocárdicas com atividade autofágica aumentada exibem menos células apoptóticas, sugerindo que a estimulação da autofagia pode prevenir a apoptose.^8^

A apoptose é necessária durante o desenvolvimento do coração e tem sido associada a muitas doenças cardiovasculares.^9^ A apoptose é mediada pela ativação de Caspases normalmente encontradas como zimogênios inativos na célula.^10^ As Caspases também podem desempenhar funções na proliferação celular, diferenciação, controle do ciclo celular e vias de sobrevivência. A via apoptótica extrínseca é iniciada pela ligação de ligantes a receptores de superfície celular que recrutam e ativam a Caspase 8, que ativa a Caspase 3 executora chave. A Caspase 3, uma das Caspases executoras mais importantes na convergência das vias apoptóticas intrínseca e extrínseca, é o principal indicador de apoptose.^11^ Como a Caspase iniciadora na via da apoptose do receptor de morte (extrínseco), a Caspase-8 ativa proteoliticamente Caspases a jusante e BID. A via intrínseca é iniciada pela liberação do citocromo c da mitocôndria. A Caspase 9, a Caspase iniciadora na via apoptótica mitocondrial (intrínseca), também participa da maioria dos processos de diferenciação nos quais a Caspase 3 foi implicada.^12 , 13^ A sinalização apoptótica nos cardiomiócitos de seres humanos é desconhecida.^13^ A apoptose dos cardiomiócitos é desencadeada por numerosos vias de sinalização e reguladas por ligantes intrínsecos e extrínsecos multicomplexos.^8 , 14 , 15^

Este estudo visa explicar como os níveis das soluções de cardioplegia Custodiol (CC) e cardioplegia sanguínea (CS) usadas para proteger o miocárdio durante a cirurgia de revascularização do miocárdio ativam os mecanismos de autofagia e apoptose ao nível do mRNA e da proteína. Assim, procuramos avaliar o dano miocárdico intraoperatório e pós-operatório imediato por autofagia e apoptose.^16^

## Material e métodos

### Seleção do paciente

**Figura 1:**
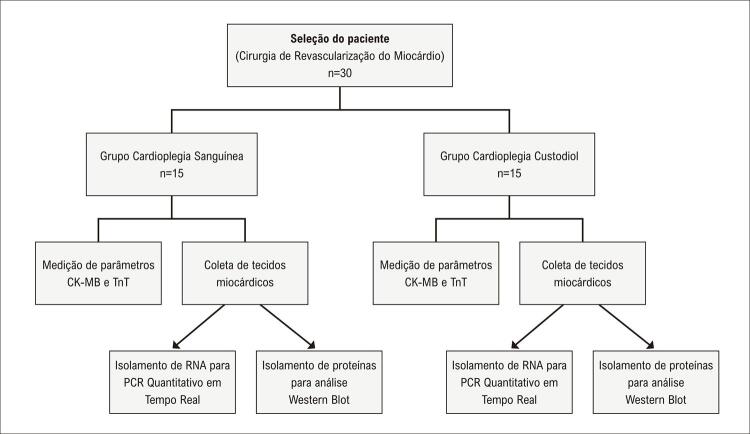
– Diagrama de fluxo da seleção do paciente no estudo. PCR: reação em cadeia da polimerase; RNA: ácido ribonucléico.

Um total de 30 pacientes com CEC foram incluídos no estudo ( Figura Central ). Este estudo é caso-controle. Os pacientes foram divididos em dois grupos de acordo com a ordem de internação. Os pacientes foram divididos em dois grupos, atentando para o tipo de procedimento cirúrgico, idade e sexo. Aplicamos CS a um grupo e CC ao outro grupo. ( Figura 1 ). Ambos os grupos consistiram em 15 pacientes. Considerando nosso trabalho anterior, o tamanho da amostra utilizado no estudo foi por conveniência.^17^ A cirurgia de revascularização miocárdica foi realizada com essas soluções cardioplégicas. Todos os pacientes estavam tomando aspirina. Nove pacientes do grupo CS e 11 pacientes do grupo CC faziam uso de β-bloqueador; 3 pacientes do grupo CS e 1 paciente do grupo CC faziam uso de bloqueadores de canais de cálcio; 4 pacientes do grupo CS e 2 pacientes do grupo CC faziam uso de medicação diabética oral; 1 paciente do grupo CS e 3 pacientes do grupo CC faziam uso de inibidor da enzima conversora de angiotensina; 1 paciente do grupo CC fazia uso de α e β bloqueadores. O grupo de controle saudável não foi usado porque os pacientes foram comparados dentro e entre os grupos. Todos os pacientes registrados pelo Departamento de Cirurgia Cardiovascular da Universidade Erciyes foram avaliados para este estudo. Pacientes com síndrome coronariana aguda, cirurgia de revascularização do miocárdio (CRM) de emergência, insuficiência renal crônica, história de cirurgia cardíaca prévia, endocardite infecciosa, doenças vasculares periféricas e doença inflamatória crônica foram excluídos do estudo. O comitê de ética da Universidade Erciyes aprovou este estudo. Formulários de consentimento informado por escrito foram recebidos dos pacientes. Este estudo respeitou as declarações éticas da Declaração de Helsinki.

**Figura Central:**
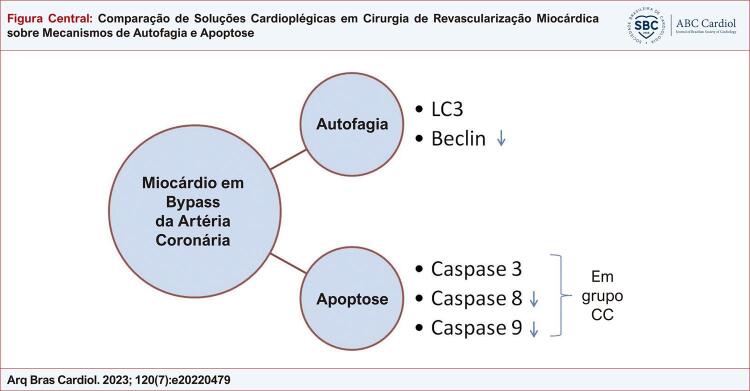
: Comparação de Soluções Cardioplégicas em Cirurgia de Revascularização Miocárdica sobre Mecanismos de Autofagia e Apoptose

### Manejo da anestesia

O mesmo protocolo de anestesia foi aplicado a todos os pacientes. Foram realizados ECG de cinco canais, oximetria de pulso, pressão arterial não invasiva, oximetria cerebral e monitoramento de entropia nos pacientes levados para a sala de cirurgia. Após injeção de 1,5 mg de dormicum e 50 microgramas de fentanil, um cateter arterial invasivo foi colocado na artéria radial e uma medição invasiva da pressão arterial foi realizada. Após pré-oxigenação, 1 mg/kg de propofol, 10 microgramas/kg de fentanil e 1 mg/kg de rocurônio foram usados na indução. Os pacientes com valores de entropia adequados foram intubados. O cateter venoso central guiado por ultrassom foi inserido. A infusão de ácido tranexâmico na dose de 15 mg/kg em 1 hora foi seguida de infusão de manutenção na dose de 1,5 mg/kg/h durante todo o caso. Dez microgramas/kg/h de fentanil, 4 mg/kg/h de propofol, e 1 mg/kg/h de rocurônio foram usados no manejo da anestesia. A profundidade da anestesia foi ajustada entre 45 e 60 de entropia; se necessário, desflurano foi usado como agente inalatório. As configurações do ventilador foram TV 6 ml/kg, frequência respiratória 12/min, PEEP de 5 cm/volume de água, com modo de controle de volume. Caso houvesse diminuição da oximetria cerebral, as intervenções necessárias eram realizadas.

### Abordagem cirúrgica

Em todos os casos, o procedimento operatório incluiu esternotomia mediana e circulação extracorpórea com oxigenador de membrana. A proteção miocárdica consistiu em hipotermia moderada (30–32°C), resfriamento tópico com solução salina fria e sangue frio ou cardioplegia Custodiol (4–10°C) administrado de forma anterógrada. A cardioplegia Custodiol [1000 cc, contendo histidina 180 (mmol/L), triptofano (2 mmol/L), manitol (30 mmol/L), KCl (9 mmol/L), NaCl (15 mMol/L)] foi administrada em uma única dose. As doses de cardioplegia sanguínea [contendo 700 cc de sangue, 2 ampolas de HCO3 (840 mg), 1 ampola de Mg (1500 mg), 2 ampolas de KCl (750 mg)] foram repetidas a cada 20 minutos. A última dose foi administrada morna (37°C) imediatamente antes da liberação do pinçamento aórtico. Todas as anastomoses distais foram realizadas primeiro, após pinçamento cruzado da aorta e infusão da solução cardioplégica. As anastomoses proximais foram feitas após a liberação do pinçamento.

### Preparação de amostras de tecido

Duas amostras de tecido miocárdico do mesmo local no apêndice atrial direito foram retiradas de cada paciente imediatamente antes do pinçamento aórtico e após a liberação da pinça (final da circulação extracorpórea). As amostras de tecido foram então congeladas em nitrogênio líquido e armazenadas a -80°C.

### Isolamento de RNA e estudos de expressão gênica

Duas amostras de tecido miocárdico (pré e pós-condicionamento) foram coletadas de cada paciente e o RNA total foi isolado com o reagente TRIZOL (Roche, Alemanha). A quantidade e a qualidade das amostras de RNA foram medidas com um Espectrofotômetro BioSpec-Nano. Os RNAs foram armazenados a -80°C até o uso. Os níveis de expressão de mRNA dos genes Beta-actina (ACTB), Beclin, LC3, Caspase 3, Caspase 8 e Caspase 9 foram determinados usando PCR em tempo real LightCycler 480 II (Roche, Alemanha). Cada amostra foi processada em duplicata. Os níveis de expressão dos genes foram calculados usando o método 2−^Ct^.

### Isolamento de proteínas e Western Blot

O isolamento de proteínas foi realizado a partir de amostras de tecido miocárdico.^16^ A concentração total de proteínas para cada amostra foi determinada com um kit de ensaio de proteína compatível com detergente (kit DC; Bio-Rad, Hercules, CA). Alíquotas contendo 40 μg de proteína total de cada amostra foram analisadas por eletroforese em gel de dodecil sulfato de sódio (SDS)-poliacrilamida com um gradiente de 4–20% para separação de proteínas e eletrotransferidas para membranas de difluoreto de polivinilideno. As membranas foram bloqueadas com um tampão de bloqueio (0,1 Triton X-100 com 5% de leite em pó em solução salina tamponada com Tris-Tween 20) [TBS-T] por 60 min. Depois de lavadas com TBS-T, as membranas foram sondadas com os seguintes anticorpos primários: LC3, Beclin-1, Caspase 3, Caspase 8 e Caspase 9 (Cell Signaling Technology). As demais etapas do western blot foram realizadas conforme indicado no estudo de Hamurcu et al.^16^ Os blots foram então visualizados com um Chemidoc MP Imaging System (Biorad) e quantificadas com um densitômetro usando o programa de aplicação de imagens (Bio-Rad Image Lab 5).

### Análise estatística

As variáveis categóricas foram apresentadas como números e porcentagens, e as comparações de grupo foram feitas usando os testes qui-quadrado, correção de Yates e Fisher. A conformidade dos dados com a distribuição normal foi avaliada com histograma, q-q plots e teste de Shapiro-Wilk. As comparações intergrupos de variáveis numéricas foram realizadas com os testes t de Student não pareados ou testes U de Mann-Whitney. A homogeneidade da variância foi avaliada com o teste de Levene. Duas amostras dependentes e o teste de Wilcoxon foram aplicados para comparar as diferenças entre os resultados de expressão gênica de mRNA e os níveis de expressão de proteína em amostras de tecido coletadas no pré e pós-operatório. Duas amostras independentes e o teste U de Mann-Whitney foram aplicados para comparar os dados do grupo cardioplegia sanguínea e cardioplegia Custodiol. Os dados foram obtidos usando GraphPad Prism 8.0. (versão 8.0.1, San Diego, CA, EUA). Valores de p <5% foram considerados estatisticamente significativos.

## Resultados

### Características do paciente e achados laboratoriais

**Figura 2:**
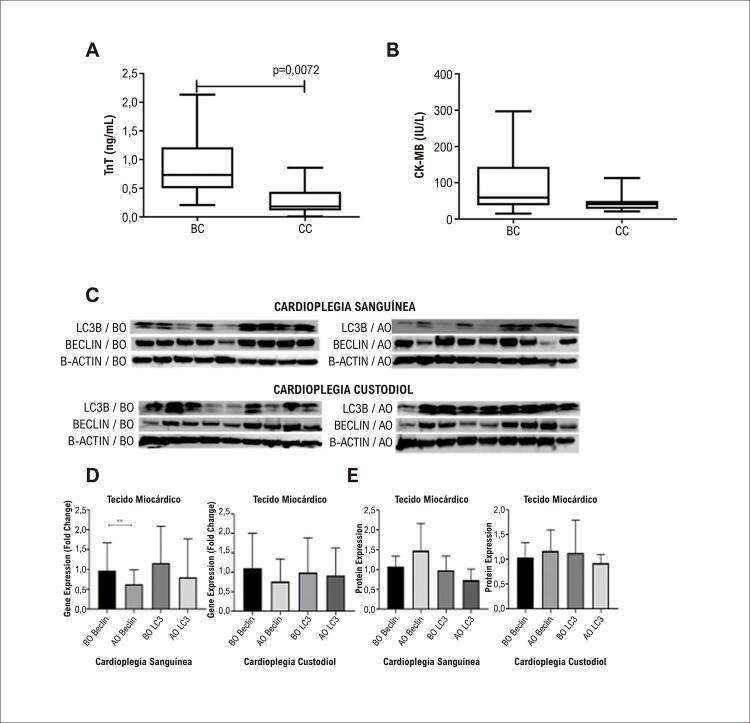
– A, B) Comparação dos achados laboratoriais pós-operatórios dos pacientes dos grupos Cardioplegia Custodiol e Cardioplegia Sanguínea. A) Os níveis de TnT foram significativamente diferenciados entre os grupos (p=0,0072). B) Os níveis de CK-MB não foram diferentes entre os grupos (p=0,0731). Figuras 2C-E) Gráficos de expressão. C) Imagens de Western Blot de membrana de marcadores de autofagia no grupo Cardioplegia Sanguínea e Cardioplegia Custodiol. D) Gráficos dos níveis de expressão dos genes Beclin e LC3 nas amostras de tecido miocárdico dos grupos (n=15, BO: Antes do pinçamento aórtico, AO: após a liberação do pinçamento aórtico). (*p<0,05). E) Expressões proteicas de Beclin e LC3 em ambos os grupos.

Quinze pacientes foram incluídos nos grupos cardioplegia sanguínea (CS) e cardioplegia Custodiol (CC). Homens (9, 60%) e mulheres (6, 40%) foram iguais em ambos os grupos. A média de idade do grupo CS foi 59,9 ± 9,8 anos, e o grupo CC foi de 52,7 ± 19,5 anos; essa diferença não foi significativa (p=0,2135). As características clínicas e cirúrgicas dos pacientes são apresentadas na Tabela 1 e os achados laboratoriais na Figura 2 (A, B) .

**Tabela 1:** – Características clínicas e demográficas do grupo de estudo

Achados clínicos	Cardioplegia sanguínea (n=15, média±DP)	Cardioplegia Custodiol (n=15, média±DP)	Valor-p
**Idade (ano médio ± SEM)**	59,9±9,8	52,7±19,5	p=0,5
Sexo (masculino n, %)	9 (60%)	9 (60%)	p=0,645
Diabetes Mellitus (n, %)	4 (27%)	2 (13%)	p=0,075
Hipertensão (n, %)	4 (27%)	2 (13%)	p=0,075
Fumante (n, %)	5 (33%)	7 (47%)	p=0,224
Altura (cm)	164±9,5	167,5±10,1	p=0,7
Peso (kg)	76,5±12,4	76,2±15	p>0,9999
ASC (m^2^)	1,8±0,2	1,8±2	p>0,9999
**Parâmetros hemodinâmicos**			
Pressão arterial sistólica	121,8 ± 16,5	123,8 ± 19,1	p=0,664
Pressão sanguínea diastólica	79,2 ± 11,6	77,7 ± 13,1	p=0,778
**Ecocardiografia**			
Fração de ejeção	54,7 ± 5,4	55,7 ± 6,3	p=0,647
Diâmetros sistólicos	3,49 ± 0,56	3,32 ± 0,3	p=0,310
Diâmetros diastólicos	5,18 ± 0,72	4,99 ± 0,44	p=0,379
Duração do Pinçamento cruzado (média min±SEM)	58,4±8,6	63,7±36,4	p=0,9931
Duração total do bypass (média min±SEM)	119±28	134,5±53,9	p=0,9423
TnT (ng/mL)	0,8 (0,5-1,6)	0,2 (0,16-1,8)	p=0,0072
CK-MB (u/L)	60,3 (39,5-145)	41 (28-50)	p=0,0731
**Cirurgia**			
CRMX1 (n)	2	2	
CRMX2 (n)	2	1	
CRMX3 (n)	6	6	
CRMX4 (n)	5	6	

CRMxN: número de by-pass; ASC: área de superfície corporal; DP: desvio padrão; SEM: erro padrão da média.

Os níveis de CK-MB e TnT foram medidos nas amostras de sangue pré e pós-operatórias do paciente para parâmetros bioquímicos e comparados nos grupos CC e CS ( Figura 2A, 2B ). Os níveis pré-operatórios de CK-MB e TnT não foram significativamente diferentes. Nossos dados mostraram que os níveis pós-operatórios de TnT diferiram significativamente entre os grupos (p=0,0072).

### Resultados de expressão gênica autofágica e apoptótica em tecido miocárdico

A expressão gênica de *BECLIN* em amostras de tecido miocárdico foi significativa e diminuiu após pinçamento aórtico no grupo CS ( Figura 2D ) (p=0,0078). A expressão do gene LC3 diminuiu após o pinçamento aórtico, mas não foi significativa ( Figura 2D ). A expressão do gene BECLIN em amostras de tecido miocárdico coletadas após o pinçamento aórtico não foi diferenciada das amostras de tecido miocárdico coletadas antes do pinçamento aórtico no grupo CC. A expressão do gene LC3 na amostra de tecido miocárdico coletada antes do pinçamento aórtico mostrou-se diminuída quando comparada à amostra de tecido miocárdico coletada após pinçamento aórtico, mas essa diminuição não foi estatisticamente significativa (p=0,7263, Figura 2E ).

**Figura 3:**
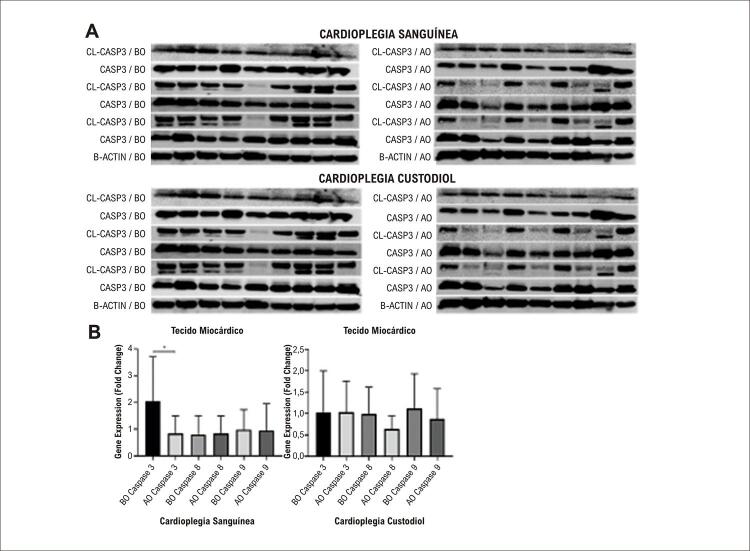
– Resultados de Western Blot. A. Imagens de Western Blot de membrana de marcadores apoptóticos no grupo Cardioplegia Sanguínea e Cardioplegia Custodiol. B. Gráficos do nível de expressão dos genes das Caspases 3, 8 e 9 nas amostras de tecido miocárdico dos grupos (n=15, BO: Antes do pinçamento aórtico, AO: após liberação do pinçamento aórtico). (*p<0,05).

A expressão de CASPASE 3 no grupo CS foi significativamente menor na amostra de tecido coletada após pinçamento aórtico (p=0,0188, Figura 3B ). As expressões dos genes CASPASE 8 e CASPASE 9 foram semelhantes antes e depois do pinçamento aórtico (p=0,8552, p=0,8891). No grupo CC, os níveis de expressão gênica de CASPASE 3, CASPASE 8 e CASPASE 9 não foram significativamente diferentes em amostras de tecido coletadas após pinçamento aórtico (p=0,7354, p=0,0758, p=0,4128, respectivamente).

Quando os níveis de expressão de mRNA de amostras de tecido miocárdico foram comparados antes e depois do pinçamento aórtico, os níveis de expressão gênica de BECLIN e LC3 não foram diferentes no grupo CS em comparação ao grupo CC ( Tabela 2 ).

**Tabela 2:** – Comparação dos níveis de expressão gênica entre amostras de tecido miocárdico de pacientes com Cardioplegia Sanguínea e Cardioplegia Custodiol

Genes	CS (BO) (n=15)	CC (BO) (n=15)	CS (AO) (n=15)	CC (AO) (n=15)	valor-p (BO/AO)
***BECLIN***	0,8(0,3-1,6)	0,6(0,5-1,4)	0,6(0,3-0,9)	0,5(0,3-1)	0,8943/ 0,8123
***LC3***	1(0,6-1,3)	0,6(0,4-1,1)	0,5(0,4-0,7)	0,6(0,3-1,4)	0,3821/ 0,6934

Os valores pré-operatórios (BO) dos grupos CS e CC foram comparados com o teste de Mann-Whitney. Os valores pós-operatórios (AO) dos grupos CS e CC foram comparados com o teste de Mann-Whitney. Os dados são expressos em mediana (1º e 3º trimestre). CS: cardioplegia sanguínea; CC: cardioplegia custodiol; BO: antes do pinçamento aórtico; AO: após liberar o pinçamento aórtico.

### Resultados do estudo de Western Blot em tecido miocárdico

O nível de expressão da proteína Beclin em amostras de tecido miocárdico coletadas após pinçamento aórtico mostrou-se aumentado, e esse valor foi estatisticamente significativo em comparação com amostras de tecido coletadas antes do pinçamento aórtico no grupo CS (p<0,05) ( Figura 2C, E ). Os níveis de expressão da proteína LC3-II diminuíram após o pinçamento aórtico, mas a diferença não foi significativa. Não houve diferença nos níveis de expressão da proteína Beclin na amostra de tecido miocárdico coletada antes do pinçamento aórtico de pacientes com CC (p>0,05), mas a expressão da proteína LC3-II foi estatisticamente significativa e diminuiu após o pinçamento aórtico (p< 0,05) ( Figura 2C, 2E ). Em ambos os grupos, os níveis de proteínas das Caspases 3, 8 e 9 clivadas diminuíram em amostras de tecido coletadas após pinçamento aórtico. Os níveis de proteína total de Caspase 3, 8 e 9 não foram alterados entre os grupos ( Figura 3A, 3B ).

## Discussão

Este estudo investigou os efeitos dos principais marcadores de autofagia e apoptose-Caspases, LC3 e Beclin, no miocárdio. Além disso, foi discutida neste estudo a questão da extensão da proteção oferecida por duas soluções cardioplégicas diferentes usadas para proteção miocárdica durante a cirurgia. Detectamos diferenças na abundância de proteínas-chave da autofagia no miocárdio atrial direito de pacientes submetidos à cirurgia de revascularização do miocárdio. Ao nosso conhecimento, este é o primeiro estudo deste tipo na literatura. Numerosos estudos estão sendo conduzidos para a manutenção da homeostase durante a cirurgia cardíaca. Os estudos mais notáveis neste campo estão relacionados a marcadores inflamatórios e vias gênicas além dos mecanismos de morte celular, como autofagia e apoptose.^8^

Os níveis de troponina e CK-MB indicam necrose e isquemia nos tecidos. A elevação desses dados clínicos também é proporcional ao grau de isquemia. Apenas uma diferença no nível de troponina foi encontrada entre os grupos a partir da comparação dos dados bioquímicos no soro (p=0,0072). Tanto a TnT quanto a CK-MB são os marcadores mais importantes de dano miocárdico. Este estudo investigou os níveis de proteção cardíaca de duas soluções de cardioplegia para determinar o dano miocárdico no pós-operatório imediato com esses marcadores. A diferença na troponina pode surgir em um pequeno número de pacientes; considera-se que o efeito exato pode ser obtido com um número maior de pacientes. Além disso, pode ser útil planejar estudos maiores para determinar a causa da diferenciação para revelar dano estrutural no miocárdio no nível de troponina.

Prathanee et al.,^18^ conduziram um estudo retrospectivo de caso-controle em pacientes com CRM. Os pacientes foram divididos em dois grupos, aplicando CS frio e solução de cardioplegia Custodiol-HTK. Houve significativamente mais fibrilação ventricular espontânea após a liberação do clampeamento cruzado no grupo Custodiol. O desfecho clínico de doses únicas de solução de CC anterógrada em cirurgia de revascularização miocárdica protegeu igualmente o CS anterógrado frio repetitivo.^18^ No total, foram identificados 1.900 procedimentos cirúrgicos cardíacos, dos quais 126 (7%) utilizaram o Custodiol e 1.774 (93%) utilizaram a CS como principal agente cardioplégico no estudo de Viana et al. Os resultados mostraram que Custodiol pode ser conveniente e pelo menos tão seguro quanto a CS para proteção miocárdica em cirurgia cardíaca complexa.^1^ Elcik et al.,^17^ mostraram que os resultados do mRNA sanguíneo deram melhores resultados na cardioplegia sanguínea. Nesse estudo, ao contrário do nosso estudo, o fato de os tempos de pinçamento serem diferentes e mais longos também podem motivar a comparação dos dados no sangue. Isso pode ser devido aos curtos tempos de clampeamento e à falta de isquemia clara completa no tecido miocárdico.^17^

A apoptose pode ser responsável por uma quantidade significativa de morte de cardiomiócitos, contribuindo para o desenvolvimento e progressão da insuficiência cardíaca. Os dados disponíveis mostram que a apoptose dos cardiomiócitos é controversa.^13 , 19^ Em cirurgia cardíaca congênita, Busro et al. usaram um grupo controle composto por 55 pacientes com apenas histidina-triptofano-cetoglutarato e um grupo de tratamento de 54 pacientes com histidina-triptofano-cetoglutarato e cardioplegia terminal de sangue quente. Uma técnica de imuno-histoquímica foi usada para examinar Caspase 3 em amostras do átrio direito. Os resultados de troponina I e Caspase-3 não diferiram significativamente entre os grupos.^20^ Dois tecidos do átrio direito foram obtidos de 24 pacientes submetidos à revascularização do miocárdio no início da enxertia e 10 minutos após a liberação da pinça aórtica. Caspase 9 e Caspase 8 ativas foram detectadas por imunocoloração para identificar as vias de indução da apoptose. Os investigadores concluíram que a parada cardioplégica com sangue quente induz apoptose de cardiomiócitos mediada por mitocôndrias.^10^ Em nosso grupo CS, a expressão da Caspase 3 diminuiu no tecido removido após pinçamento aórtico, e esse dado foi estatisticamente significativo. No grupo CC, as expressões de Caspase 8 e Caspase 9 diminuíram após o pinçamento aórtico, mas isso foi insignificante. Isso indica que o CC suprime a morte celular apoptótica e acredita-se que proteja contra a morte celular apoptótica. Além disso, pensamos que a via intrínseca pode ser desencadeada no grupo CS, e ambas as vias intrínseca e extrínseca são desencadeadas no grupo CC. Conforme resumido acima, não há estudos na literatura comparando as soluções cardioplégicas utilizadas para proteção miocárdica em nível molecular. Este é o primeiro estudo a investigar o mecanismo da apoptose no tecido miocárdico, tanto no nível do transcriptoma quanto no nível da proteína.

Garcia et al. estudaram se a autofagia atrial é ativada em pacientes que desenvolvem fibrilação atrial (FAPO) pós-operatória. LC3B foi acentuadamente reduzido em pacientes com FAPO. A autofagia cardíaca prejudicada está presente em pacientes que desenvolvem FAPO após cirurgia de revascularização do miocárdio.^21^ Foi demonstrado que LC3 e BECLIN não foram diferenciados no tecido miocárdico ventricular esquerdo de pacientes submetidos à cirurgia de circulação extracorpórea. Quando o mecanismo de autofagia está avançado, as proteínas autofágicas também são degradadas, explicando potencialmente a depleção de proteínas autofágicas durante CEC mais longa.^7^ Apoiando essa informação, os níveis de proteína e transcrição de proteínas autofágicas (Beclin-1 e LC3-II) também diminuíram na falha miocárdio do ventrículo esquerdo (VE) de pacientes com cardiomiopatia dilatada idiopática após explantação de um dispositivo de assistência do VE.^22^ Foi relatado na literatura que o mecanismo de autofagia desempenha um papel significativo na proteção do miocárdio. Este estudo examinou os mRNAs e a expressão de proteínas de Caspases, LC3 e Beclin no tecido do miocárdio durante a cirurgia de revascularização do miocárdio para determinar quaisquer diferenças entre as duas soluções cardioprotetoras (Cardioplegia sanguínea versus cardioplegia Custodiol). Nos grupos submetidos à cardioplegia sanguínea e à cardioplegia Custodiol, foi feita uma comparação na proteção do miocárdio via mecanismo de autofagia. As expressões dos genes LC3 e Beclin não foram estatisticamente significativas em ambos os grupos neste estudo. Além disso, de acordo com o resultado do Western Blot, a expressão da proteína Beclin foi maior nos grupos de CS e CC. Além disso, Beclin tem um papel anti-autofágico na interação com BCL-2; portanto, pode nem sempre atuar com LC3. Afirma-se também que Beclin tem um papel protetor contra doenças cardíacas em outros mecanismos dentro da célula (supressão tumoral, desenvolvimento, imunidade), além de seu papel na autofagia.^23 , 24^ Além disso, descobriu-se que Beclin é armazenado em diferentes áreas dentro do celular.^22^ Assim, podemos considerar que o aumento da expressão da proteína é um indicador de que a Beclin está armazenada na célula, embora a expressão do mRNA da Beclin tenha diminuído no pós-operatório. Não houve diferença quanto à proteção miocárdica na comparação entre as duas soluções cardioplégicas. Nem os grupos de cardioplegia de sangue nem de Custodiol mostraram qualquer diferença na proteção miocárdica em relação aos marcadores autofágicos.

A busca na literatura indicou que o número de estudos sobre amostras de miocárdio colhidas durante a cirurgia é escasso. O número de estudos realizados com transcriptoma e Western Blot é muito pequeno. Além disso, não há nenhum estudo sobre o uso e comparação de duas soluções cardioplégicas diferentes semelhante a este estudo. Portanto, este estudo se diferencia de outros devido a esses aspectos. Nossos achados foram relacionados à proteção miocárdica no pós-operatório imediato. Seria conveniente planejar novos estudos que suportassem os resultados de nosso estudo com um número maior de pacientes nos grupos. A maior limitação deste estudo é o pequeno número de pacientes. Os resultados podem ser diferentes com um estudo multicêntrico. A segunda grande limitação são os curtos tempos de pinçamento cruzado. Longos tempos de pinçamento exigirão cardioplegia adicional, e esses resultados podem variar.

## Conclusão

Este estudo pode iluminar estudos futuros sobre o tratamento cirúrgico das cardiopatias e contribuir para o desenvolvimento científico no tratamento dessas doenças. A autofagia tem efeitos bidirecionais como mecanismo de morte celular. Não encontramos nenhuma diferença entre as soluções CS e CC usadas neste estudo ao examinar a morte ou sobrevivência celular. Por isso, acreditamos que ambas as soluções cardioprotetoras podem ser utilizadas durante as operações, e os resultados do nosso estudo podem fazer a diferença em futuras cirurgias cardíacas com esse método.
