# Supplementary material for: Comparação de Soluções Cardioplégicas em Cirurgia de Revascularização Miocárdica sobre Mecanismos de Autofagia e Apoptose
Source: Arq Bras Cardiol. 2023 Jun 28;120(7):e20220479. [Article in Portuguese] doi: 10.36660/abc.20220479 (PMC10364987; doi:10.36660/abc.20220479)
Supplement: Translation

# Translation: Comparison of Cardioplegic Solutions in Coronary Bypass Surgery Over Autophagy and Apoptosis Mechanisms

**Authors:** Elif Funda Sener, Zuhal Hamurcu, Serpil Taheri, Reyhan Tahtasakal, Nesrin Delibasi, Deniz Elcik, Ecmel Mehmetbeyoglu, Aydin Tuncay, Fatma Dal, Keziban Korkmaz Bayram, Isın Gunes, Omer Naci Emirogullari

**Affiliations:** 1 Turkey Erciyes University Medical Faculty Department of Medical Biology, Kayseri – Turkey; 2 Turkey Erciyes University Genome and Stem Cell Center (GENKOK), Kayseri – Turkey; 3 Turkey Cappadocia University Cappadocia Vocational College Department of Medical Laboratory Techniques, Nevsehir – Turkey; 4 Turkey Erciyes University Medical Faculty Department of Cardiology, Kayseri – Turkey; 5 Turkey Erciyes University Medical Faculty Department of Cardiovascular Surgery, Kayseri – Turkey; 6 Turkey Ankara Yildirim Beyazit University Medical Faculty Department of Medical Genetics, Ankara – Turkey; 7 Turkey Erciyes University Medical Faculty Department of Anesthesiology and Reanimation, Kayseri – Turkey

**Keywords:** Coronary Artery Disease, Myocardial Revascularization, Myocardial Ischemia, Cardioplegic Solutions, Autophagy, Apoptosis

## Abstract

**Background:**

Coronary artery disease (CAD) due to myocardial ischemia causes permanent loss of heart tissue.

**Objectives:**

We aimed to demonstrate the possible damage to the myocardium at the molecular level through the mechanisms of autophagy and apoptosis in coronary bypass surgery patients.

**Methods:**

One group was administered a Custodiol cardioplegia solution, and the other group was administered a Blood cardioplegia solution. Two myocardial samples were collected from each patient during the operation, just before cardiac arrest and after the aortic cross-clamp was released. The expressions of autophagy and apoptosis markers were evaluated. The level of statistical significance adopted was 5%.

**Results:**

The expression of the BECLIN gene was significant in the myocardial tissues in the BC group (p=0.0078). CASPASE 3, 8, and 9 gene expression levels were significantly lower in the CC group. Postoperative TnT levels were significantly different between the groups (p=0.0072). CASPASE 8 and CASPASE 9 gene expressions were similar before and after aortic cross-clamping (p=0.8552, p=0.8891). In the CC group, CASPASE 3, CASPASE 8, and CASPASE 9 gene expression levels were not found to be significantly different in tissue samples taken after aortic cross-clamping (p=0.7354, p=0.0758, p=0.4128, respectively).

**Conclusions:**

With our findings, we believe that CC and BC solutions do not have a significant difference in terms of myocardial protection during bypass operations.

## Introduction

Cardioplegia solutions are used to arrest the heart and reduce ischemic damage in the myocardium when coronary blood flow is stopped during cardiac operations.^1 , 2^ Since Custodiol and blood cardioplegia are highly effective solutions, they have been used for a long time in open-heart surgeries. During cardiopulmonary bypass (CPB), the heart is arrested and protected with cardioplegia solutions. This period is associated with oxygen deprivation; the heart is ischemic during CPB. The heart is reperfused at the end of CPB, and cardiac action continues.^3^ However, CPB may lead to further myocardial damage caused by global reperfusion and cell death through the induction of myocardial autophagy and apoptosis.^4^

Autophagy is thought to play an absolute role in heart tissue during ischemia/reperfusion. While induction of autophagy in cardiomyocytes during ischemia has a protective effect, autophagy induced during reperfusion is thought to lead to cardiomyocyte death. Under normal conditions, metabolic stress typically stimulates apoptosis. However, in cells where the mechanism of apoptosis is impaired, the cell continues to live under hypoxic conditions. The survival of these cells is due to autophagy, but when both apoptosis and autophagy are suppressed, cell viability fails and dies.^5^ Autophagy occurs at basal levels but can be further induced by stress, such as hypoxia and nutrient depletion.^6^ The protein LC3B is an important player in the autophagic process. The activation of autophagy is reflected by increases in the abundance of key proteins of the autophagy-related genes (ATG5-12), light chain 3 (LC3), Beclin-1, and p62.^7^ Myocardial regions with increased autophagic activity exhibit fewer apoptotic cells, suggesting that the stimulation of autophagy may prevent apoptosis.^8^

Apoptosis is required during heart development and has long been associated with many cardiovascular diseases.^9^ Apoptosis is mediated by activating caspases normally found as inactive zymogens in the cell.^10^ Caspases can also play roles in cell proliferation, differentiation, cell cycle control, and survival pathways. The extrinsic apoptotic pathway is initiated by binding ligands to cell surface receptors that recruit and activate Caspase 8, which activates the key executioner Caspase 3. Caspase 3, one of the most important executioner caspases in the convergence of the intrinsic and extrinsic apoptotic pathways, is the main indicator of apoptosis.^11^ As the initiator caspase in the death receptor (extrinsic) apoptosis pathway, Caspase 8 proteolytically activates downstream caspases and BID. The intrinsic pathway is initiated by cytochrome c release from the mitochondrion. Caspase 9, the initiator caspase in the mitochondrial (intrinsic) apoptotic pathway, also participates in most differentiation processes in which caspase 3 has been implicated.^12 , 13^ Apoptotic signaling in the cardiomyocytes of human subjects is unknown.^13^ Cardiomyocyte apoptosis is triggered by numerous signaling pathways and regulated by multi-complex intrinsic and extrinsic ligands.^8 , 14 , 15^

This study aims to explain how levels of Custodiol cardioplegia (CC) and blood cardioplegia (BC) solutions used to protect the myocardium during coronary bypass surgery activate the mechanisms of autophagy and apoptosis at the level of mRNA and protein. Thus, we tried to evaluate intraoperative and early postoperative myocardial damage through autophagy and apoptosis.^16^

## Material and Methods

### Patient Selection

A total of 30 CBP patients were included in the study ( Central Illustration ). This study is case-controlled. Patients were divided into two groups according to the order of hospitalization. The patients were divided into two groups paying attention to the type of operation procedure, age, and gender. We applied BC to one group and CC to the other group. ( Figure 1 ). Both groups consisted of 15 patients. Considering our previous work, the sample size used in the study was for convenience.^17^ Coronary bypass surgery was performed with these cardioplegic solutions. All patients were taking aspirin. Nine patients in the BC group and 11 patients in the CC group were using β-blocker; 3 patients in the BC group and 1 patient in the CC group were using Ca channel blockers; 4 patients in the BC group and 2 patients in the CC group were taking oral diabetic medication; 1 patient in the BC group and 3 patients in the CC group were using angiotensin-converting enzyme inhibitor; 1 patient in the CC group was using α and β blockers. The healthy control group was not used because the patients were compared within and between the groups. All patients registered by the Erciyes University Department of Cardiovascular Surgery were evaluated for this study. Patients with acute coronary syndrome, emergency coronary artery bypass grafting (CABG), chronic renal failure, previous heart surgery history, infective endocarditis, peripheral vascular diseases, and chronic inflammatory disease were excluded from the study. The ethical committee of Erciyes University approved this study. Written informed consent forms were received from the patients. This study complied with the ethical statements in the Helsinki Declaration.

**Central Illustration:** : Comparison of Cardioplegic Solutions in Coronary Bypass Surgery Over Autophagy and Apoptosis Mechanisms

**Figure 1:** – Flow diagram of the patient selection in the study. PCR: polymerase chain reaction; RNA: ribonucleic acid.

### Anesthesia management

The same anesthesia protocol was applied to all patients. Five-channel ECG, pulse oximetry, noninvasive blood pressure, cerebral oximetry, and entropy monitoring were performed on the patients taken to the operating room. After 1.5 mg dormicum and 50 microgram fentanyl push, an invasive arterial catheter was placed in the radial artery, and an invasive blood pressure measurement was performed. After preoxygenation, 1 mg/kg propofol, 10 micrograms/kg fentanyl, and 1 mg/kg rocuronium were used in the induction. Patients with adequate entropy values were intubated. The ultrasound-guided central venous catheter was inserted. Tranexamic acid infusion at a dose of 15 mg/kg in 1 hour was followed by maintenance infused at 1.5 mg/kg/h throughout the case. Ten micrograms/kg/h fentanyl, 4 mg/kg/h propofol, and 1 mg/kg/h rocuronium were used in anesthesia management. The depth of anesthesia was adjusted between 45 and 60 entropy; if necessary, desflurane was used as an inhalation agent. Ventilator settings were TV 6 ml/kg, respiratory rate 12/min, PEEP of 5 cm/water volume, with volume control mode. If there was a decrease in cerebral oximetry, necessary interventions were performed.

### Surgical approach

In all cases, the operative procedure included median sternotomy and cardiopulmonary bypass with a membrane oxygenator. Myocardial protection consisted of moderate hypothermia (30–32°C), topical cooling with cold saline solution, and cold blood or CC (4–10°C) administered in an antegrade fashion. CC [1000 cc, containing histidine 180 (mmol/L), tryptophan (2 mmol/L), mannitol (30 mmol/L), KCl (9 mmol/L), NaCl (15 mMol/L)] was given in a single dose. BC [containing 700 cc blood, 2 ampules of HCO_3_(840 mg), 1 ampule of Mg (1500 mg), 2 ampules of KCl (750 mg)] doses were repeated every 20 minutes. The last dose was given warm (37°C) just before releasing the aortic cross-clamp. All distal anastomoses were performed first, after cross-clamping the aorta and infusion of the cardioplegic solution. The proximal anastomoses were done after the release of the cross-clamp.

### Tissue sample preparation

Two myocardial tissue samples from the same site on the right atrial appendage were taken from each patient immediately before aortic cross-clamping and after releasing the clamp (end of the cardiopulmonary bypass). Tissue samples were then frozen in liquid nitrogen and stored at -80°C.

### RNA isolation and gene expression studies

Two myocardial tissue samples (pre and post-conditioning) were taken from each patient, and total RNA was isolated using TRIZOL reagent (Roche, Germany). The quantity and quality of RNA samples were measured with a BioSpec-Nano Spectrophotometer. RNAs were stored at −80°C until use. mRNA expression levels of Beta-actin ( *ACTB* ), *Beclin, LC3, Caspase 3, Caspase 8,* and *Caspase 9* genes were determined using LightCycler 480 II (Roche, Germany) Real-Time PCR. Each sample was run in duplicate. The expression levels of the genes were calculated by using the 2−^Ct^method.

### Protein isolation and Western Blot

Protein isolation was performed from myocardial tissue samples.^16^ The total protein concentration for each sample was determined with a detergent-compatible protein assay kit (DC kit; Bio-Rad, Hercules, CA). Aliquots containing 40 μg of total protein from each sample were analyzed by sodium dodecyl sulfate (SDS)-polyacrylamide gel electrophoresis with a 4–20% gradient for protein separation and electrotransferred to polyvinylidene difluoride membranes. The membranes were blocked with a blocking buffer (0.1 Triton X-100 with 5% dry milk in Tris-buffered saline–Tween 20) [TBS-T] for 60 min. After being washed with TBS-T, the membranes were probed with the following primary antibodies: LC3, Beclin-1, Caspase 3, Caspase 8, and Caspase 9 (Cell Signaling Technology). The remaining stages of the western blot were performed as stated in the study of Hamurcu et al.^16^ The blots were then visualized with a Chemidoc MP Imaging System (Biorad) and quantified with a densitometer using the imager application program (Bio-Rad Image Lab 5).

### Statistical analysis

Categorical variables were presented as numbers and percentages, and group comparisons were performed using chi-square, Yates correction, and Fisher tests. The conformity of the data to the normal distribution was evaluated with histogram, q-q plots, and Shapiro-Wilk test. Intergroup comparisons of numerical variables were performed with the unpaired Student t-tests or Mann-Whitney U tests. The homogeneity of variance was evaluated with Levene’s test. Two dependent samples and the Wilcoxon test were applied to compare the differences between mRNA gene expression results and protein expression levels in tissue samples taken pre and post-operatively. Two independent samples and the Mann-Whitney U test were applied to compare BC and CC group data. Data were performed using GraphPad Prism 8.0. (Version 8.0.1, San Diego, CA, USA) software. P values of <5% were considered statistically significant.

## Results

### Patient characteristics and laboratory findings

Fifteen patients were included in the BC (BC) and CC groups. Males (9, 60%) and females (6, 40%) were equal in both groups. The mean age of the BC group was 59.9 ± 9.8 years, and the CC group was 52.7 ± 19.5 years; this difference was not found significant (p=0.2135). Clinical and surgical characteristics of the patients are shown in Table 1 , and laboratory findings are shown in Figure 2 (A, B) .

**Table 1:** – Clinical and demographical features of the study group

Clinical Findings	Blood Cardioplegia (n=15, mean±SD)	Custodiol Cardioplegia (n=15, mean±SD)	p-value
**Age (mean year±SEM)**	59.9±9.8	52.7±19.5	p=0.5
Gender (Male n,%)	9 (60%)	9 (60%)	p=0.645
Diabetus Mellitus (n,%)	4 (27%)	2 (13%)	p=0.075
Hypertension (n,%)	4 (27%)	2 (13%)	p=0.075
Smoking (n,%)	5 (33%)	7 (47%)	p=0.224
Height (cm)	164±9.5	167.5±10.1	p=0.7
Weight (kg)	76.5±12.4	76.2±15	p>0.9999
BSA (m^2^)	1.8±0.2	1.8±2	p>0.9999
**Hemodynamic parameters**			
Systolic blood pressure	121.8 ± 16.5	123.8 ± 19.1	p=0.664
Diastolic blood pressure	79.2 ± 11.6	77.7 ± 13.1	p=0.778
**Echocardiography**			
Ejection fraction	54.7 ± 5.4	55.7 ± 6.3	p=0.647
Systolic Diameters	3.49 ± 0.56	3.32 ± 0.3	p=0.310
Diastolic Diameters	5.18 ± 0.72	4.99 ± 0.44	p=0.379
Cross Clamp duration (mean min±SEM)	58.4±8.6	63.7±36.4	p=0.9931
Total bypass duration (mean min±SEM)	119±28	134.5±53.9	p=0.9423
TnT (ng/mL)	0.8 (0.5-1.6)	0.2 (0.16-1.8)	p=0.0072
CK-MB (u/L)	60.3 (39.5-145)	41 (28-50)	p=0.0731
**Surgery**			
CABGX1 (n)	2	2	
CABGX2 (n)	2	1	
CABGX3 (n)	6	6	
CABGX4 (n)	5	6	

CABGxN: Number of bypass GRAFT; BSA: Body surface area; SD: standard deviation; SEM: standard error of mean.

**Figure 2:** – A, B) Comparison of the postoperative laboratory findings of the patients in Custodiol Cardioplegia and Blood Cardioplegia groups. A) TnT levels were significantly differentiated between the groups (p=0.0072). B) CK-MB levels were not different between the groups (p=0.0731). Figures 2C-E) Expression graphs. C) Western blot membrane images of autophagy markers in Blood Cardioplegia and Custodiol Cardioplegia group. D) Beclin and LC3 gene expression level graphs in myocardial tissue samples in the groups (n=15, BO: Before aortic cross-clamping, AO: after releasing aortic cross-clamping). (*p<0.05). E) Protein expressions of Beclin and LC3 in both groups.

CK-MB and TnT levels were measured in the patient’s preoperative and postoperative blood samples for biochemical parameters and compared in CC and BC groups ( Figure 2A, 2B ). Preoperative levels of CK-MB and TnT were not significantly different. Our data showed that postoperative TnT levels significantly differed between the groups (p=0.0072).

### Autophagic and apoptotic gene expression results in myocardial tissue

*BECLIN* gene expression in myocardial tissue samples was significant and decreased after aortic cross-clamping in the BC group ( Figure 2D ) (p=0.0078). *LC3* gene expression decreased after aortic cross-clamping but was insignificant ( Figure 2D ). *BECLIN* gene expression in myocardial tissue samples taken after aortic cross-clamping was not differentiated from myocardial tissue samples taken before aortic cross-clamping in the CC group. *LC3* gene expression in the myocardial tissue sample taken before aortic cross-clamping was found to be decreased when compared to the myocardial tissue sample taken after aortic cross-clamping, but this decrease was not statistically significant (p=0.7263, Figure 2E ).

The expression of *CASPASE 3* in the BC group was significantly lower in the tissue sample taken after aortic cross-clamping (p=0.0188, Figure 3B ). *CASPASE 8* and *CASPASE 9* gene expressions were similar before and after aortic cross-clamping (p=0.8552, p=0.8891). In the CC group, *CASPASE 3, CASPASE 8,* and *CASPASE 9* gene expression levels were not found to be significantly different in tissue samples taken after aortic cross-clamping (p=0.7354, p=0.0758, p=0.4128, respectively).

**Figure 3:** – Western Blot Results. A) Western blot membrane images of apoptotic markers in Blood Cardioplegia and Custodiol Cardioplegia group. B) Caspase 3, 8, and 9 gene expression level graphs in myocardial tissue samples in the groups (n=15, BO: Before aortic cross-clamping, AO: after releasing aortic cross-clamping). (*p<0.05).

When mRNA expression levels of myocardial tissue samples were compared before and after aortic cross-clamping, *BECLIN* and *LC3* mRNA gene expression levels were not different in the BC group compared to the CC group ( Table 2 ).

**Table 2:** – Comparison of gene expression levels between myocardial tissue samples from patients with Blood Cardioplegia and Custodiol Cardioplegia

Genes	BC (BO) (n=15)	CC (BO) (n=15)	BC (AO) (n=15)	CC (AO) (n=15)	p-value (BO/AO)
***BECLIN***	0.8(0.3-1.6)	0.6(0.5-1.4)	0.6(0.3-0.9)	0.5(0.3-1)	0.8943/ 0.8123
***LC3***	1(0.6-1.3)	0.6(0.4-1.1)	0.5(0.4-0.7)	0.6(0.3-1.4)	0.3821/ 0.6934

The BC and CC groups’ preoperative values (BO) were compared with the Mann-Whitney test. The BC and CC groups’ postoperative values (AO) were compared with the Mann-Whitney test. Data are expressed as median (1st and 3rd quarter). BC: blood cardioplegia; CC: custodiol cardioplegia; BO: before aortic cross-clamping; AO: after releasing aortic cross-clamping.

### Western Blot Study results in myocardial tissue

Beclin protein expression level in myocardial tissue samples taken after aortic cross-clamping was found to be increased, and this value was statistically significant compared to tissue samples taken before aortic cross-clamping in the BC group (p<0.05) ( Figure 2C, E ). LC3-II protein expression levels were found to be decreased after aortic cross-clamping, but the difference was not significant. There was no difference in the expression levels of Beclin protein in the myocardial tissue sample taken before aortic cross-clamping of CC patients (p>0.05), but LC3-II protein expression was statistically significant and decreased after aortic cross-clamping (p<0.05) ( Figure 2C, 2E ). In both groups, the protein levels of cleaved Caspases 3, 8, and 9 were found to be decreased in tissue samples taken after aortic cross-clamping. Total Caspase 3, 8, and 9 protein levels were not changed between the groups ( Figure 3A, 3B ).

## Discussion

This study investigated the effects of the main autophagy and apoptosis markers-caspases, LC3 and Beclin, on the myocardium. Additionally, the question regarding the extent of protection provided by two different cardioplegic solutions used for myocardial protection during surgery was discussed in this study. We detected differences in the abundance of key autophagy proteins in the right atrial myocardium of patients undergoing coronary bypass surgery. To our knowledge, this is the first study of this type in the literature. Numerous studies are being conducted for the continuation of homeostasis during heart surgery. The most remarkable studies in this field are related to inflammatory markers and gene pathways other than cell death mechanisms such as autophagy and apoptosis.^8^

Troponin and CK-MB levels indicate necrosis and ischemia within tissues. The elevation of such clinical data is also proportional to the degree of ischemia. Only a difference in the troponin level was found between the groups from the comparison of biochemical data in serum (p=0.0072). Both TnT and CK-MB are the most important markers of myocardial damage. This study investigated the heart protection levels of two cardioplegia solutions to determine myocardial damage in the early postoperative period with these markers. The difference in troponin may arise in a small number of patients; it is considered that the exact effect may be obtained with a larger number of patients. In addition, it may be useful to plan larger and different studies for determining the cause of differentiation to reveal structural damage on the myocardium at the troponin level.

Prathanee et al.^18^ conducted a retrospective case-control study in CABG patients. The patients were divided into two groups, applying cold BC and Custodiol-HTK cardioplegia solution. There was significantly more spontaneous ventricular fibrillation after cross-clamping was released in the Custodiol group. The clinical outcome of single doses of antegrade CC solution in CABG surgery protected myocardium equally and repetitive antegrade cold BC.^18^ In total, 1.900 cardiac surgical procedures were identified, of which 126 (7%) utilized Custodiol and 1.774 (93%) used BC as the primary cardioplegic agent in Viana et al.’s study. The results showed that Custodiol might be convenient and at least as safe as BC for myocardial protection in complex cardiac surgery.^1^ Elcik et al.^17^ showed that blood mRNA results gave better results in BC. In this study, unlike our study, the fact that the cross-clamp times were different and longer may also be the comparison of the data in the blood. This may be due to the short cross-clamp times and lack of complete clear ischemia in myocardial tissue.^17^

Apoptosis may be responsible for a significant amount of cardiomyocyte death, contributing to heart failure’s development and progression. The available data have shown that cardiomyocyte apoptosis is controversial.^13 , 19^ In congenital cardiac surgery, Busro et al. used a control group consisting of 55 patients with only histidine-tryptophan-ketoglutarate and a treatment group of 54 patients with histidine-tryptophan-ketoglutarate and terminal warm BC. An immunohistochemistry technique was used to examine Caspase 3 in right atrial samples. The results of troponin I and Caspase-3 did not differ significantly between the groups.^20^ Two right atrium tissues were obtained from 24 patients undergoing coronary artery bypass grafting at the start of grafting and 10 minutes after the release of the aortic clamp. Active Caspase 9 and Caspase 8 were detected by immunostaining to identify the pathways of apoptosis induction. The investigators concluded that warm blood cardioplegic arrest induces mitochondria-mediated cardiomyocyte apoptosis.^10^ In our BC group, Caspase 3 expression decreased in the tissue removed after aortic cross-clamp, and this data was statistically significant. In the CC group, Caspase 8 and Caspase 9 expressions decreased after the aortic cross-clamp, but this was insignificant. This indicates that CC suppresses apoptotic cell death and is thought to protect against apoptotic cell death. Besides, we think the intrinsic pathway may be triggered in the BC group, and both intrinsic and extrinsic pathways are triggered in the CC group. As summarized above, no studies in the literature compared the cardioplegic solutions used for myocardial protection at the molecular level. This is the first study to investigate the mechanism of apoptosis in myocardial tissue at both transcriptome and protein levels.

Garcia et al. studied whether atrial autophagy is activated in patients who develop postoperative atrial fibrillation (POAF). LC3B was markedly reduced in POAF patients. Impaired cardiac autophagy is present in patients developing POAF after coronary artery bypass surgery.^21^ It was shown that LC3 and *BECLIN* were not differentiated in left ventricular myocardial tissue from patients undergoing cardiopulmonary bypass surgery. When the autophagy mechanism is advanced, autophagic proteins are also degraded, potentially explaining the depletion of autophagic proteins during longer CPB.^7^ Supporting this information, protein and transcript levels of autophagic proteins (Beclin-1 and LC3-II) were also decreased in failing left ventricular (LV) myocardium of patients with idiopathic dilated cardiomyopathy after explantation of an LV assist device.^22^ It was reported in the literature that the autophagy mechanism plays a significant role in the protection of the myocardium. This study examined mRNAs and protein expression of Caspases, LC3, and Beclin in myocardial tissue during coronary bypass surgery to determine any differences between the two cardioprotective solutions (BC versus CC). In groups undergoing BC and CC, a comparison was made in the myocardium protection via the autophagy mechanism. The *LC3* and *Beclin* gene expressions were not statistically significant in both groups in this study. Moreover, according to the western blot result, Beclin protein expression was higher in the groups of BC and CC. Furthermore, Beclin has an anti-autophagic role in interaction with BCL-2; therefore, it may not always act with LC3. It is also stated that Beclin has a protective role against cardiac diseases in other mechanisms within the cell (tumor suppression, development, immunity) besides its role in autophagy.^23 , 24^ Also, it was found that Beclin is stored in different areas within the cell.^22^ Thus, we may consider that the increased protein expression is an indicator that Beclin is stored in the cell even though the mRNA expression of Beclin decreased post-surgery. No difference was observed regarding myocardium protection in comparing the two cardioplegic solutions. Neither blood nor CC groups showed any difference in myocardial protection regarding autophagic markers.

The literature search indicated that the number of studies on myocardium samples taken during surgery is scarce. The number of studies conducted with transcriptome and western blot is very small. Besides, there is no study on the usage and comparison of two different cardioplegic solutions similar to this study. Therefore, this study is differentiated from other studies due to these aspects. Our findings were related to myocardial protection in the early postoperative period. It would be suitable to plan new studies supporting the results of our study with a larger number of patients in the groups. The biggest limitation of this study is the small number of patients. Results may be different with a multicenter study. The second major limitation is the short cross-clamp times. Long cross-clamp times will require additional cardioplegia, and these results may vary.

## Conclusion

This study may enlighten future studies on the surgical treatment of heart diseases and contribute to scientific developments for treating such diseases. Autophagy has bidirectional effects as a cell death mechanism. We found no difference between BC and CC solutions used in this study from examining cell death or survival. For this reason, we believe that both cardioprotective solutions may be used during operations, and our study results may make a difference in future heart surgeries with this method.
